# Small Bowel Obstruction Secondary to Migration of Tubal Ligation Clips: A Case Report

**DOI:** 10.7759/cureus.83241

**Published:** 2025-04-30

**Authors:** Max J Green, Binura B Lekamalage, Avinash S Sharma

**Affiliations:** 1 General Surgery, Whakatane Hospital, Whakatane, NZL

**Keywords:** adhesions, bowel obstruction, clip migration, gastrografin, tubal ligation

## Abstract

A 72-year-old female presented with acute abdominal pain, vomiting, and obstipation, suggestive of a small bowel obstruction (SBO). Her surgical history included an open tubal ligation performed over four decades earlier. On examination, the patient had a distended abdomen with tenderness, particularly in the right lower quadrant. Computed tomography (CT) revealed multiple dilated small bowel loops with a transition point in the pelvis. Although tubal ligation clips were observed on CT in this region, they were not initially considered a contributing factor. Conservative management failed to resolve the obstruction, leading to the need for emergent laparotomy. Intraoperatively, two tubal ligation clips were found to have eroded into the small bowel, creating an adhesional band resulting in the SBO. The clips had also induced a bowel stricture, necessitating resection and subsequent anastomosis. This case and the accompanying images illustrate a rare cause of SBO due to the migration of tubal ligation clips. This emphasizes the importance of a comprehensive surgical history and the potential for clip migration to contribute to adhesional SBO, which may be resistant to conservative treatment.

## Introduction

Small bowel obstruction (SBO) is a common pathology in general surgery. In New Zealand, it accounts for approximately 20% of acute admissions to the service [1]. Post-operative adhesions are the leading cause of SBOs, accounting for 65% of cases [2,3]. Peritoneal adhesions are aberrant fibrous bands that develop among abdomino-pelvic organs and limit physiologic visceral motion of the bowel, resulting in obstruction [4]. Alternative aetiologies are encompassed by hernias (10%), neoplasms (5%), Crohn's disease (5%), and other rarer causes such as volvulus, gallstone ileus, obstructive foreign bodies, and bezoars (15%) [3]. The majority of these cases, around 65-80%, resolve with non-operative management [5,6]. We present an unusual case of SBO caused by migration of tubal ligation clips placed 42 years prior, which required operative management.

## Case presentation

A 72-year-old female presented to a rural New Zealand hospital with a two-day history of acute abdominal pain, vomiting, and obstipation. Her surgical history included open tubal ligation with combined appendicectomy in 1982. Her medical history included hypertension, hypercholesterolaemia, gastroesophageal reflux disease, and chronic obstructive pulmonary disease. On examination, the patient had a distended abdomen with generalised tenderness worse in the right lower quadrant. Initial biochemical investigations were unremarkable.

**Figure 1:**
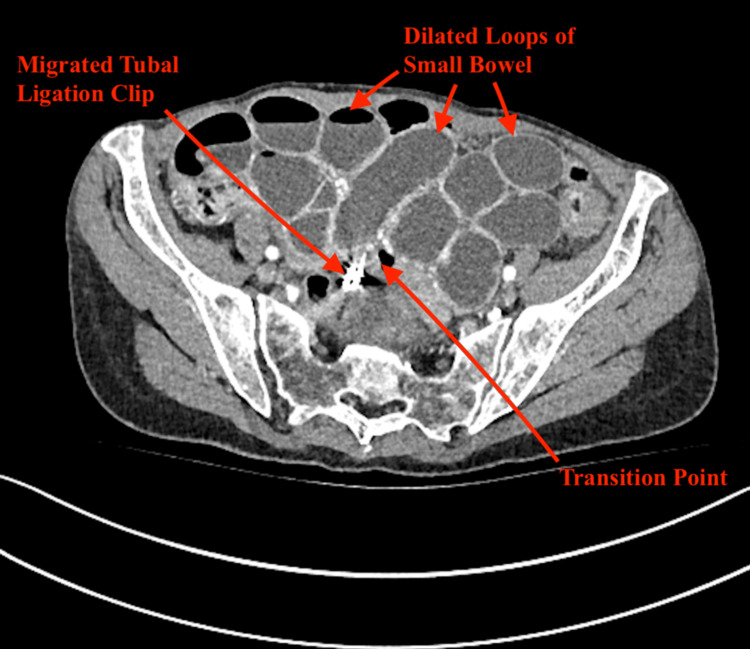
Contrast-enhanced CT of the abdomen demonstrating multiple dilated small bowel loops. The transition point is closely associated with a migrated tubal ligation clip.

A computed tomography (CT) of the abdomen with intravenous (IV) contrast demonstrated multiple dilated small bowel loops (maximally 36 mm in diameter) with a transition point in the pelvis suggestive of an adhesive SBO and a tubal ligation clip within the region of concern (Figure 1). She was managed conservatively for the initial 48 hours with IV fluids, nasogastric decompression, and a trial of Gastrografin. However, repeated imaging demonstrated an absence of contrast within the colon. Having failed conservative management, there was concern for complete obstruction and the potential for bowel compromise, and the decision was made to take the patient to theatre.

**Figure 2:**
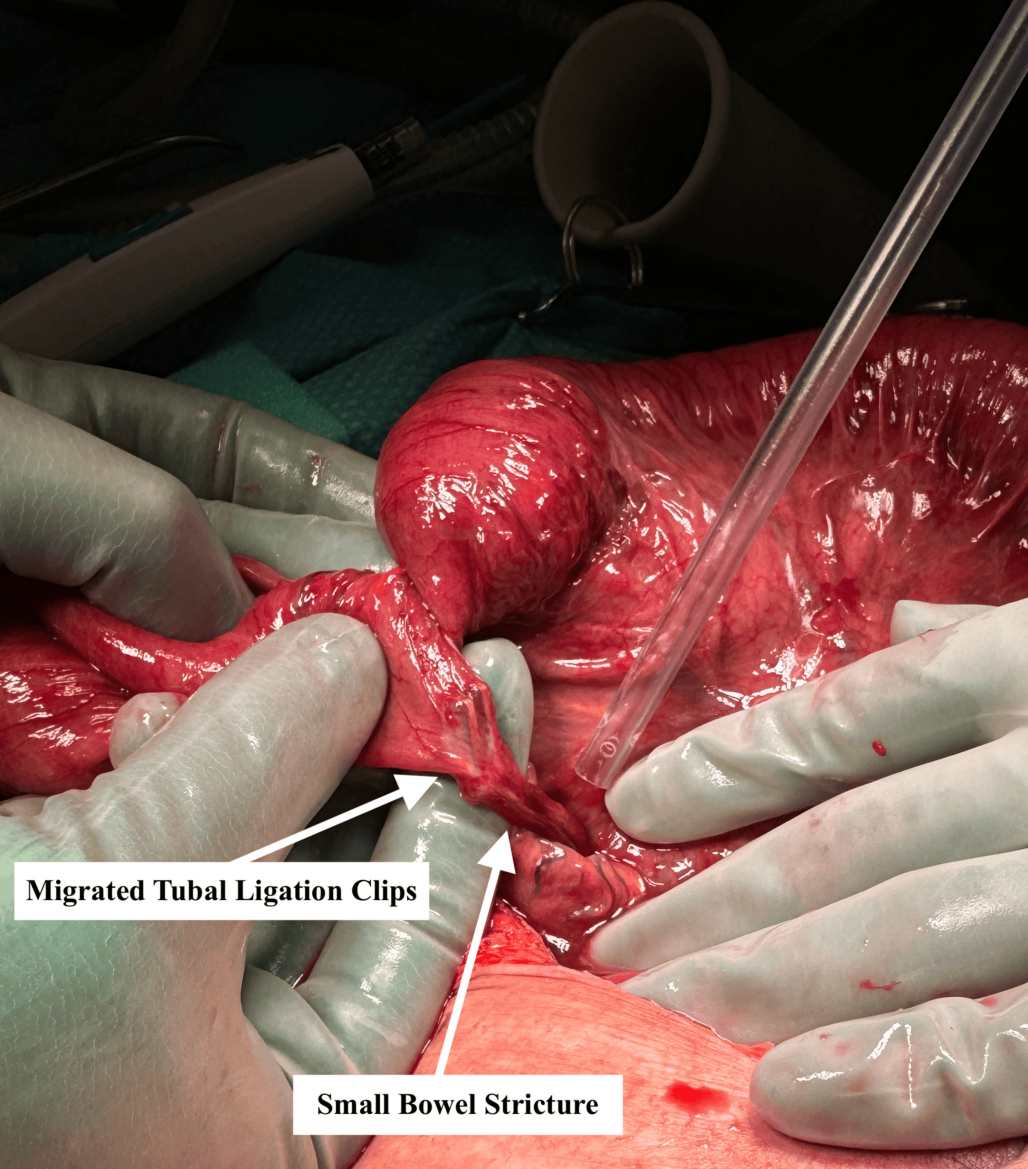
Intraoperative image showing a tubal ligation clip eroding into the small bowel with an associated stricture.

The patient proceeded to theatre on day three of admission for emergent diagnostic laparoscopy. Intra-operatively, two migratory clips from prior tubal ligation were seen to have eroded into the small bowel, with an associated adhesional band creating a transition point. A small bowel enterotomy was also noted at this time. The small bowel was friable and significantly distended, consistent with strangulation, and the decision was made to convert to laparotomy (Figure 2). The adhesional band was divided to relieve the obstruction; however, it became evident that the erosion of the clips had created a stricture. The strictured portion of the small bowel was resected, and a side-to-side iso-peristaltic double-stapled anastomosis was performed. The enterotomy was managed with a two-layer closure. This was followed by a thorough washout of the peritoneal cavity, placement of a 15 French (Fr) Blake drain (Ethicon, Johnson & Johnson, USA), and commencement of IV cefuroxime and metronidazole. The patient’s postoperative course was complicated by prolonged ileus; however, she was successfully discharged home 14 days after surgery.

## Discussion

This case represents a rare cause of SBO due to migrated tubal ligation clips, a phenomenon not previously reported in the literature. Tubal ligation is considered a safe and effective method of permanent contraception [7]. Between 1983 and 1986, 22% of New Zealand women reported having had tubal ligation [8]. The rate of permanent contraception has remained relatively stable since this time; however, the nature of the procedure is changing with greater favour for bilateral salpingectomy [9,10]. This corresponds with emerging evidence that ovarian cancer originates in the fallopian tubes and removal offers a decrease in ovarian cancer risk beyond laparoscopic tubal ligation [10]. This trend also decreases the risk posed by leaving foreign bodies, such as tubal ligation clips, within the abdomen, where they can migrate, causing complications [11].

Migration of tubal ligation clips is not uncommon, reportedly 25% of cases; however, only 1% are clinically significant [11]. Migration of clips predominantly causes minor complications, including pain or localised infection [11,12]. There are a number of cases in the literature reporting SBO secondary to aberrant surgical instruments after a variety of surgical interventions. These include instruments intentionally placed within the abdomen, such as clips and staples, or unintentionally retained surgical instruments like sponges and gauze [13-15]. These previously recorded cases of SBO due to surgical foreign bodies tended to occur in close temporal proximity to the index operation. This case highlights that the complication of SBO can occur even after an extended period of time post-operatively, with this presentation occurring more than 40 years later. All of the aforementioned cases required surgical intervention over conservative management, demonstrating the importance of recognising this as an aetiology of SBO. The potential for SBO to occur due to aberrant surgical instruments underscores the importance of a comprehensive surgical history when evaluating patients with acute abdominal conditions concerning for obstruction [16]. This case adds to the history of complications caused by instruments generally regarded as benign. Although cases are rare, with the increasing incidence of laparoscopic surgery and the increasingly complex surgical instruments utilised, the potential harm of leaving foreign material within the abdomen should be considered, particularly in younger patients who have more years over which complications may arise. A history of tubal ligation or the use of other surgical clips and staples may indicate a patient population more likely to fail conservative management [11].

A challenging question for a treating team is when to operate on an SBO. The most feared complication of SBO is strangulation, leading to ischemia, necrosis, and ultimately perforation and sepsis [13]. Recognition of SBO cases that will resolve with non-surgical management is challenging, and many articles have addressed this issue [5]. The Bologna guidelines present an evidence-based approach to the diagnosis and treatment of adhesional SBO [5]. The guidelines recommend a trial of non-operative management in all patients with adhesive SBO unless there are signs of peritonitis, strangulation, or bowel ischemia. In determining if the cause of bowel obstruction is adhesive, the recommendation is for CT, ideally with oral water-soluble contrast, to exclude other causes and identify signs of ischemia or strangulation. Once the cause is established and the need for urgent surgical exploration is excluded, the cornerstone of non-operative management is insertion of a naso-gastric tube for decompression, with the patient kept nil by mouth [5]. The use of water-soluble contrast studies, such as Gastrografin, can aid decision-making, demonstrating patients unlikely to resolve with conservative treatment [17]. This prognostication is important with the average hospitalisation after surgical treatment of SBO being 16 days, compared to five days following non-operative treatment. There is, of course, also considerable risk for bowel injury from emergency surgical exploration as well as associated high morbidity [5]. Unsuccessful resolution after 24-36 hours, marked by lack of contrast in the colon, is indicative of a need for operative intervention [5]. A large proportion of these patients will have features of strangulation detected intraoperatively, as was seen in this case [18].

## Conclusions

This case underscores the importance of considering the possibility of aberrant surgical instruments contributing to SBO in any patient presenting with a history of abdominal surgery, regardless of time from the index operation. This patient population may be more likely to fail conservative management due to the nature of the obstruction. It highlights the importance of consideration when leaving surgical foreign bodies in patients, particularly if they are young, as avoidance of which may help prevent a future obstruction. A thorough surgical history is critical in the evaluation of patients with unexplained abdominal symptoms, as early recognition of unusual causes like clip migration can guide timely and appropriate management. This case demonstrates that migration of tubal ligation clips, while uncommon, can result in serious complications such as bowel erosion, adhesional bands, and strictures, leading to persistent obstruction requiring surgical intervention.
